# Barriers to Practice and the Impact on Health Care: A Nurse Practitioner Focus

**Published:** 2017-01-01

**Authors:** Mary E. Peterson

**Affiliations:** From St. David’s South Austin Medical Center, Austin, Texas

In 1965, in answer to the growing demand for primary care, nursing pioneer Loretta Ford, along with Dr. Henry Silver, created the first certificate program that provided nurses with the skills to provide primary care to underserved populations. Since the inception of that first program, the field has grown to include adult/gerontology, women’s health, neonatal, and other specialty roles, with minimum requirements for education at the master’s/doctoral level.

The growth of nurse practitioners (NPs) across all 50 states is unsurprising given the current landscape of health care, yet barriers that limit practice need action at both the state and national levels. Access to care is a significant challenge for patients. This access is negatively impacted when qualified NPs are willing and able to deliver quality, cost-effective care, yet governmental bodies continue to ignore legislation that would update the laws and modernize health care.

## IDENTIFYING RESTRICTIONS

Individual states regulate NP practice. Currently, 22 states and the District of Columbia, or 44%, have adopted full practice authority licensure and practice laws for NPs. Full practice authority is defined by the American Association of Nurse Practitioners (AANP) as follows: "State practice and licensure law provides for all nurse practitioners to evaluate patients, diagnose, order and interpret diagnostic tests, initiate and manage treatments—including prescribe medications—under the exclusive licensure authority of the state board of nursing." The remaining states are categorized as either "reduced practice" (17 states, or 34%) or "restricted practice" (12 states, or 24%). The AANP further defines these categories as follows (AANP, 2016):

*Reduced Practice*: The NP has the ability to engage in at least one element of the NP practice and is regulated through a collaborative agreement with an outside health discipline to provide patient care.

*Restricted Practice*: The NP has the ability to engage in at least one element of NP practice and requires supervision, delegation, or team management by an outside health discipline to provide patient care.

In 2010, the Institute of Medicine (IOM), with the Robert Wood Johnson Foundation (RWJF), published a landmark report titled, "The Future of Nursing: Leading Change, Advancing Health." This paper outlined four key messages: (1) Nurses should practice to the full extent of their education; (2) nurses should achieve higher levels of education and training through an improved education system that promotes seamless academic progression; (3) nurses should be full partners, with physicians and other health-care professionals, in redesigning health care in the United States; and (4) effective work force planning and policy making require better data collection and an improved information infrastructure (IOM, 2010).

The report gives recommendations for implementing the key messages and recognizes "overly restrictive scope-of-practice regulation of NPs in some states as one of the most serious barriers to accessible care" (IOM, 2010). Soon after the release of this report, the RWJF, with the American Association of Retired Persons (AARP), launched the Future of Nursing: Campaign for Action (the Campaign), which has since worked at the national and state levels to shepherd the report’s recommendations.

As a follow-up to the 2010 report, in 2015 RWJF released the report, "Assessing Progress on the Institute of Medicine Report: The Future of Nursing," which states that the Campaign had made significant progress in a short period of time, but points out that barriers still exist and more work needs to be done. The report adds that we need to continue to "address challenges in the areas of health care delivery and scope of practice, education, collaboration, leadership, diversity in the nursing profession, and work force data" (National Academies of Sciences, Engineering, and Medicine, 2015).

## SCOPE-OF-PRACTICE REGULATIONS

Despite the original report in 2010, we still struggle against some of the same barriers. In some states, the battle to practice within the scope-of-practice regulations becomes further impeded by archaic hospital bylaws. Nurse practitioners with the same educational preparation and national certification may face a host of restrictions when relocating from one state to another, thus limiting their scope of practice (Safriet, 2011). Variations in the scope-of-practice regulations across states have an indirect impact on patient care, as the degree of physician supervision may affect practice opportunities and payer polices for NPs (Yee et al., 2013).

Further research looks promising in regard to health-care costs. One such study in 2013 "was inconclusive for total health-care spending" (Martsolf et al., 2015). However, it did show that prices appear to go down slightly while utilization increases due to improvements in access to care. Spending in nine states that grant NPs full prescriptive authority does seem to increase slightly for some services, such as office visits, but acute coronary syndrome (ACS)-related emergency visits tend to drop.

Nurse practitioner independence might reorient spending toward higher-value services. If, as the studies suggest, full practice authority of NPs leads to more office-based primary care visits and checkups and fewer ACS emergency visits, then value per dollar spent should increase. There is not enough evidence to know the answer definitively. It does appear that restrictive laws could, in some states, force NPs to pay a significant share of practice revenues to their collaborating physicians.

Federal regulations place additional barriers on NP practice despite some small victories. The Balanced Budget Act of 1997 included the Primary Care Health Practitioner Incentive Act, perhaps the most important payment reform to affect NPs. Although the Primary Care Health Practitioner Incentive Act allowed for NPs to bill for services, one of the remaining challenges is the continued existence of the two-level fee structure. Nurse practitioners can receive 100% reimbursement for incident to services, but they cannot do so independently, thereby placing another barrier for the formation of independent practices and access to health care.

Many arguments against full practice cite safety as a concern. Evidence regarding the impact of NPs compared with physicians (MDs) on health-care quality, safety, and effectiveness has been systematically reviewed by Stanik-Hutt et al. (2013). Data from 37 of 27,993 articles published from 1990 to 2009 were summarized into 11 aggregated outcomes. According to the authors, "Outcomes for NPs compared to MDs (or teams without NPs) are comparable or better for all 11 outcomes reviewed".

## UPDATE ON RESTRICTIVE STATES

Regardless of the overwhelming amount of data and evidence in support of full practice authority, many states continue to deny NPs the right to practice to the full extent of their license. According to the AANP (2016), the 12 restrictive states are California, Florida, Georgia, Massachusetts, Michigan, Missouri, North Carolina, Oklahoma, South Carolina, Tennessee, Texas, and Virginia.

Texas falls at the lower end of the spectrum regarding the freedoms it offers NPs. Despite legislative initiatives such as the Nurse Practitioner Full Practice Authority Bill (HB 1885) sponsored by Representatives Rodriguez and Burton at the 84th Legislative Session in 2015, the billed failed to make it to committee due to pressure from members of the State Medical Board. In many circles, especially health care in Texas, the term "good ole boy" may still ring true, reinforcing these barriers.

One misconception is that NPs want to broaden our scope of practice. We are simply trying to practice to the extent of our licensure. Specifically, in Texas, our scope is defined by the Board of Nursing as follows: "The advanced nurse practitioner acts independently and/or in collaboration with the health team in the observation, assessment, diagnosis, intervention, evaluation, rehabilitation, care and counsel, and health teachings of persons who are ill, injured or infirm, or experiencing changes in normal health processes; and in the promotion and maintenance of health or prevention of illness..." (21 Texas Administrative Code 221.13).

While many gains are promising, getting legislators to hear our bills can often be a daunting task. Many state NP organizations have hired lobbyists to help bring their efforts to the front line of legislators, although the medical boards in these states have significant influence and financial power to push their agendas through. It’s time to change the focus from "us against them" to providing patients in the communities we serve with access to quality care.

"Instead of drawing divisive lines based on our training or credentials, we ought to find ways to identify and improve the poor performers in each group, without constraining the stars. Concluding that all doctors are great and all nurses just average is as misguided as judging someone’s intellect by the college he or she attended" (Prasad, 2016). We need to adopt partnerships within the medical profession that support our initiatives and continue to present data that support full practice authority.

The National Council of State Boards of Nursing (NCSBN) tracks the practice roles and related bills for all advanced practice registered nurses. The Table represents initiatives in restrictive authority states as previously outlined. The NCSBN attempts to maintain current active logs; however, all updates may not be represented in the Table. According to Maureen Cahill of the NCSBN, some gains were made in 2016 in both restrictive and nonrestrictive states—such as Virginia (clinical nurse specialist [CNS] role), West Virginia (autonomy after a transition period), Tennessee (title), Alaska (title and license), and Washington (CNS role)—but the fight is far from over.

**Table:**
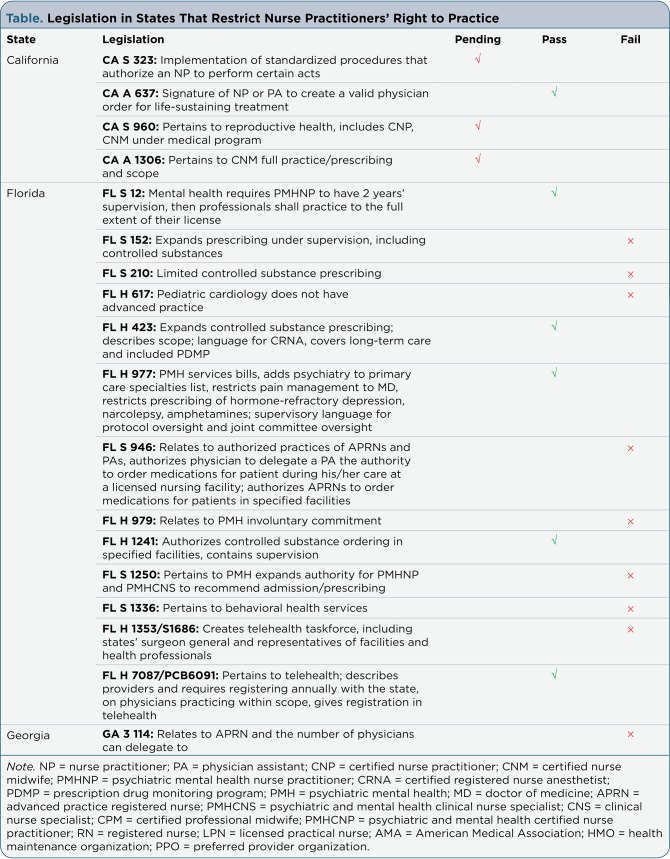
Legislation in States That Restrict Nurse Practitioners’ Right to Practice

**Table:**
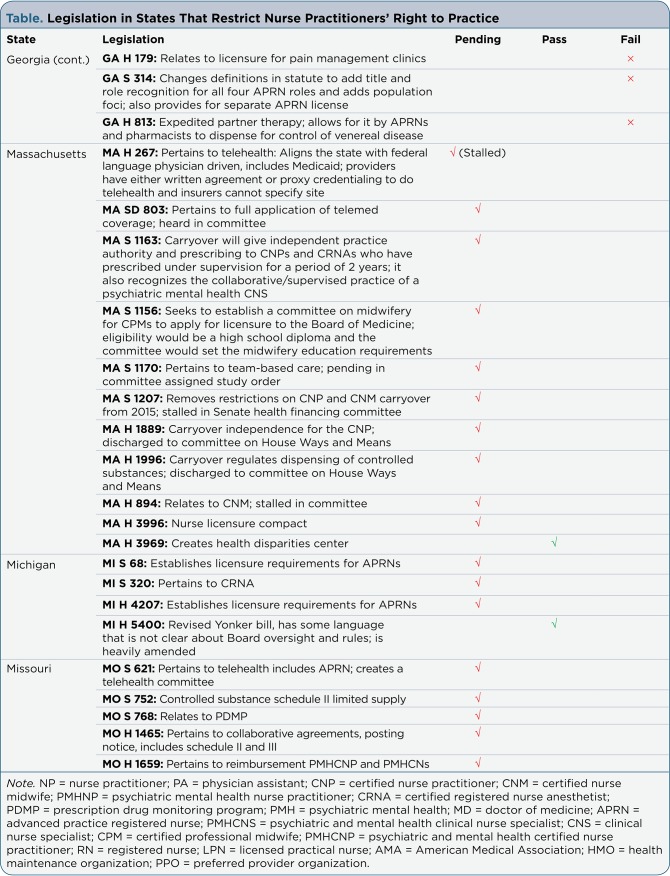
Legislation in States That Restrict Nurse Practitioners’ Right to Practice (cont.)

**Table:**
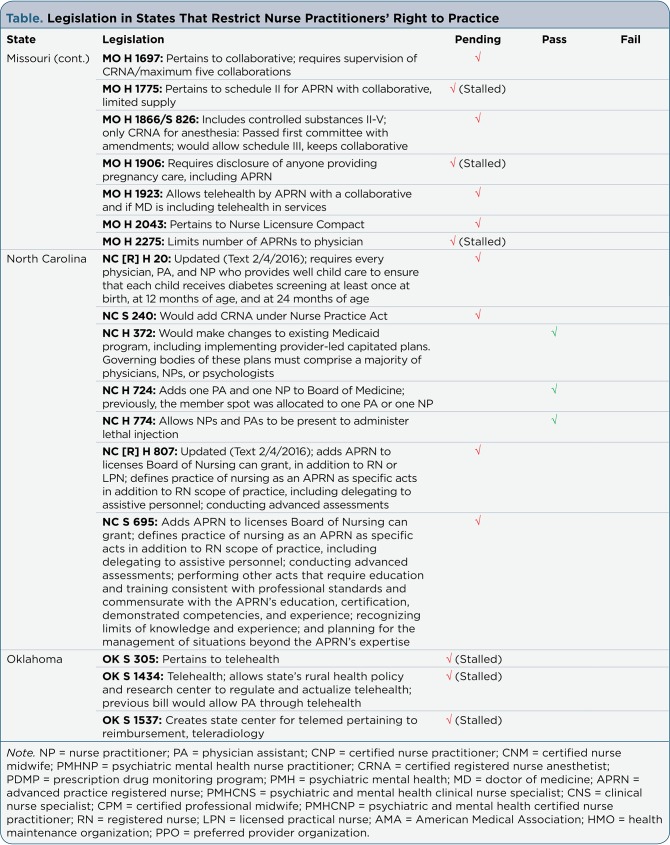
Legislation in States That Restrict Nurse Practitioners’ Right to Practice (cont.)

**Table:**
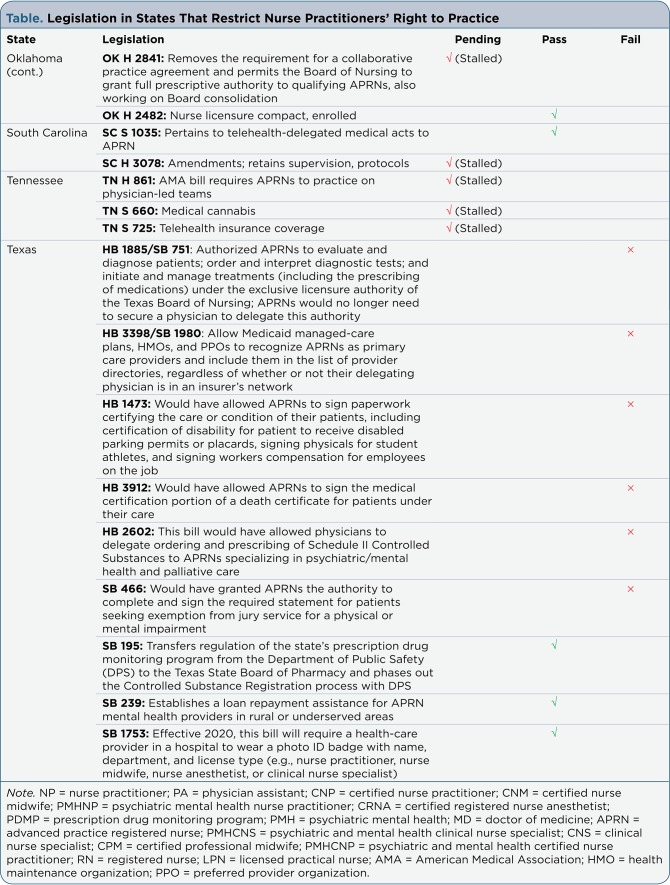
Legislation in States That Restrict Nurse Practitioners’ Right to Practice (cont.)

**Table:**
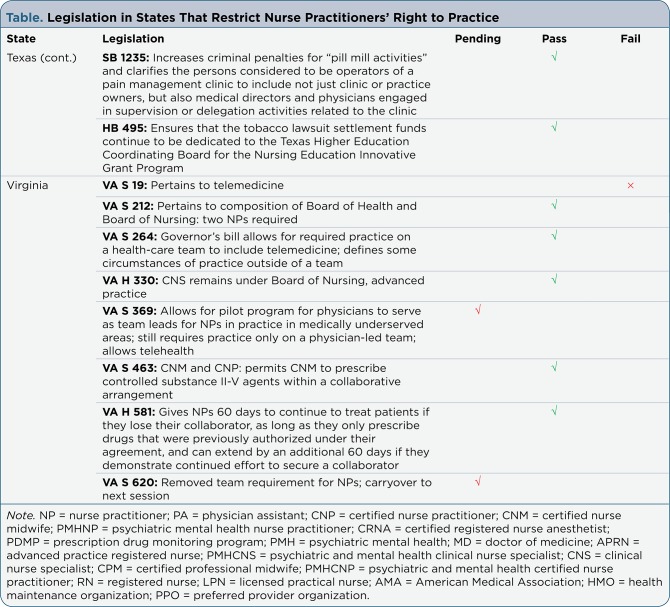
Legislation in States That Restrict Nurse Practitioners’ Right to Practice (cont.)

It will take dedicated, determined leaders to help bring about change. As one of my colleagues, Erin Perez, NP, recently stated in an interview on full practice: "Do you want to own your practice, or are you just renting space?"

## References

[1] American Association of Nurse Practitioners (AANP). (2016). State practice environment.. https://www.aanp.org/legislation-regulation/state-legislation/state-practice-environment.

[2] Bankston K, Glazer G (2013). Legislative: Interprofessional collaboration: What’s taking so long?. *The Online Journal of Issues in Nursing*.

[3] Hain D, Fleck L M (2014). Barriers to NP practice that impact healthcare redesign.. *The Online Journal of Issues in Nursing*.

[4] Institute of Medicine (IOM). (2010). *The future of nursing: Leading change, advancing health.*.

[5] Institute of Medicine (IOM). (2013). *Interprofessional education for collaboration: Learning how to improve health from interprofessional models across the continuum of education to practice: Workshop summary.*.

[6] Institute of Medicine (IOM). (2015). *Measuring the impact of interprofessional education on collaborative practice and patient outcomes.*.

[7] Interprofessional Education Collaborative Expert Panel. (2011). * Core competencies for interprofessional collaborative practice: Report of an expert panel.*.

[8] Martsolf G R, Auerbach D I, Arifkhanova A (2015). The impact of full practice authority for nurse practitioners and other advanced practice registered nurses in Ohio.. http://www.rand.org/pubs/research_reports/RR848.html.

[9] National Academies of Sciences, Engineering, and Medicine. (2015). *Assessing progress on the Institute of Medicine Report: The future of nursing.*.

[10] Prasad V (2016). Let nurse practitioners work independently.. https://www.statnews.com/2016/05/24/nurse-practitioners-doctors/.

[11] Safriet B J (2011). Federal options for maximizing value of advanced practice nurses in providing quality, cost-effective health care.. *The future of nursing: Leading change, advancing health.*.

[12] Stanik-Hutt J, Newhouse R P, White K M, Johantgen M, Bass E B, Zangaro G, Weiner J P (2013). The quality and effectiveness of care provided by nurse practitioners.. *Journal for Nurse Practitioners*.

[13] Sullivan M, Kiovsky R D, Mason D J, Hill C D, Dukes C (2015). Interprofessional collaboration and education: Working together to ensure excellence in health care.. *American Journal of Nursing*.

[14] US Department of Health and Human Services (HHS), Health Resources and Services Administration, National Center for Health Workforce Analysis. (2014). *The future of the nursing workforce: National- and state-level projections, 2012-2025.*.

[15] Yee T, Boukus E, Cross D, Samuel D (2013). Primary care workforce shortages: Nurse practitioner scope-of-practice laws and payment policies.. http://nihcr.org/wp-content/uploads/2015/03/NIHCR_Research_Brief_No._13.pdf.

